# Correction: Nanoscale protein architecture of the kidney glomerular basement membrane

**DOI:** 10.7554/eLife.01663

**Published:** 2013-10-11

**Authors:** Hani Suleiman, Lei Zhang, Robyn Roth, John E Heuser, Jeffrey H Miner, Andrey S Shaw, Adish Dani

Suleiman H, Zhang L, Roth R, Heuser JE, Miner JH, Shaw AS, Dani A. 2013. Nanoscale protein architecture of the kidney glomerular basement membrane. *eLife* **2**:e01149. doi: 10.7554/eLife.01149. Published 08 October 2013

Jeffrey H Miner was not originally listed as a corresponding author. The article has been corrected accordingly.

